# Therapeutic Effects of Acupuncture through Enhancement of Functional Angiogenesis and Granulogenesis in Rat Wound Healing

**DOI:** 10.1155/2012/464586

**Published:** 2012-12-10

**Authors:** Sang In Park, Yun-Young Sunwoo, Yu Jin Jung, Woo Chul Chang, Moon-Seo Park, Young-An Chung, Lee-So Maeng, Young-Min Han, Hak Soo Shin, Jisoo Lee, Sang-Hoon Lee

**Affiliations:** ^1^Institute of Catholic Integrative Medicine (ICIM), Incheon St. Mary's Hospital, The Catholic University of Korea, Incheon 403-720, Republic of Korea; ^2^Department of Radiology, Incheon St. Mary's Hospital, The Catholic University of Korea, Incheon, Republic of Korea; ^3^Chicago Medical School, Rosalind Franklin University of Medicine and Science, North Chicago, USA; ^4^Department of Radiology, St. Mary's Hospital, The Catholic University of Korea, Seoul, Republic of Korea

## Abstract

Acupuncture regulates inflammation process and growth factors by increasing blood circulation in affected areas. In this study, we examined whether acupuncture has an effect on wound healing in injured rat. Rats were assigned randomly into two groups: control group and acupuncture group. Acupuncture treatment was carried out at 8 sites around the wounded area. We analyzed the wound area, inflammatory cytokines, proliferation of resident cells, and angiogenesis and induction of extracelluar matrix remodeling. At 7 days after-wounding the wound size in acupuncture-treat group was decreased more significantly compared to control group. In addition, the protein levels of proinflammatory cytokines such as tumor necrosis factor-**α** (TNF-**α**) and interleukin-1**β** (IL-1**β**) were significantly decreased compared to the control at 2 and 7 days post-wounding. Also, we analyzed newly generated cells by performing immunostaining for PCNA and using several phenotype markers such as CD-31, **α**-SMA, and collagen type I. In acupuncture-treated group, PCNA-positive cell was increased and PCNA labeled CD-31-positive vessels, **α**-SMA- and collagen type I-positive fibroblastic cells, were increased compared to the control group at 7 days post-wounding. These results suggest that acupuncture may improve wound healing through decreasing pro-inflammatory response, increasing cell proliferation and angiogenesis, and inducing extracellular matrix remodeling.

## 1. Introduction

In normal wound repair, well-controlled and coordinated balance between immune defense and epithelial cell proliferation and differentiation is essential. Wound healing involves a complex process that includes inflammation, proliferation, epithelialization, angiogenesis, and collagen matrix formation [1, 2]. During the inflammatory phase of skin repair, neutrophils and macrophages infiltrate the wounded area to clear damaged tissue and produce cytokines such as macrophage inflammatory protein (MIP)-1*α*, interleukin (IL) family, and tumor necrosis factor-*α* (TNF-*α*) to expedite the repair process [3]. Epithelialization occurs by migration and proliferation of keratinocytes from the wound edges and by differentiation of stem cells from the remaining hair follicle bulbs [4, 5]. Also, collagen deposition occurs by the influx of growth factors such as transforming growth factor-*β*, fibroblast growth factor (FGF), and vascular endothelial growth factor (VEGF) that are secreted by macrophages, platelets, and fibroblasts. Moreover, angiogenesis is a multistep process that involves endothelial cell sprouting from the parent vessel, by migration, proliferation, alignment, and tube formation to other vessels [6, 7]. Angiogenesis plays important roles in the healing process of wound [7–9]. This process is associated with the expression of angiogenic factors such VEGF and platelet derived growth factor (PDGF). 

Acupuncture is an Asian medical procedure with a long history that involves peripheral sensory stimulation for treatment of various ailments. Recently, a number of studies tried to elucidate the physiology of acupuncture. Our previous study demonstrated that acupuncture on specific acupoints induces changes of glucose metabolism in the brain [10]. Furthermore, acupuncture attenuates inflammation increasing blood circulation at the affected area [11]. Some studies reported therapeutic effect of acupuncture on Ashi point. As we know, Ashi-point is pain site that is found on the body by the practitioner. Since Ashi points can be anywhere, there is an unlimited number of them. Sun group demonstrated that there is similar therapeutic effect of acupuncture site between acupoint and Ashi-point in orthopedic postoperative pain [12]. Lee et al. and Nakajima et al. reported that acupuncture treatment in wound and around bone fracture accelerates healing process by reducing proinflammation and stimulating epidermal regeneration [13, 14]. Therefore, we believe acupuncture may provide a non-toxic and natural alternative to wound healing. However, the exact mechanisms behind the effects of acupuncture are still not clear.

In this study, we investigated whether acupuncture treatment around the edges of wound improved wound healing and promoted the proliferation of resident cells in wounded area of rats. Also, we studied the effects of acupuncture on angiogenesis and induction of extracelluar matrix remodeling and change of pro-inflammatory cytokines in wound area.

## 2. Methods

### 2.1. Animals

The experimental protocol used in this study was designed in compliance with the guidelines established by the Institutional Animal Care and Use Committee of Catholic University Medical School. Male Spraque-Dawley rats (270–300 g) were initially anesthetized with 5% isoflurane in 70% nitrous oxide and 30% oxygen using an induction chamber, and were maintained by a mixture of 2% isoflurane under temperature controlled conditions (37 ± 0.1°C) using a rectal thermometer and heating pad (Harvard Apparatus Inc., Holliston, Massachusetts, USA). After shaving and cleaning with 70% ethanol, the dorsal skin was picked up at the midline and punched through two layer of skin (10 mm in diameter). The animals were divided into two groups: (1) inhalation anesthesia group after wound (*n* = 7, Control group) and (2) acupuncture-treated experimental group (*n* = 7, acupuncture group). Each group was subdivided into three time point group (2 and 7 days after wound; *n* = 6 for each time point). 

### 2.2. Acupuncture Treatment Procedure

**Figure 1:**
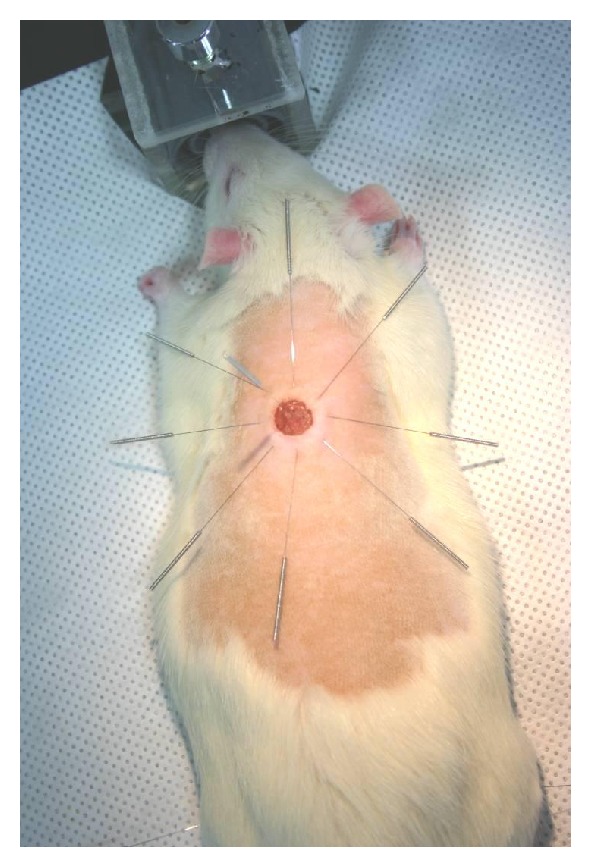
Acupuncture treatment after wound. Image obtained at 1 day after wound showing wounded skin being treated with acupuncture.

The acupuncture treatment was given daily for 20 min at 9 sites in wound area, by using a stainless press-needle of 0.25 mm in diameter and 30 mm in length (Suzhou Hua Tuo Medical Instruments Co. Ltd Suzhou, China). The rats were maintained under inhalation anesthesia in thermally regulated conditions (37 ± 0.1°C) using a rectal thermometer and heating pad (Harvard Apparatus Inc., Holliston, Massachusetts, USA). The acupuncture needles were placed and fixed on the skin around the wound (Figure 1). Needles were accurately inserted to the targeted depth of 1.5 mm and were sustained with 20 min per day for 5 days after wound. 

### 2.3. Wound Closure Measurements

Immediately after creating the wounds, the initial wound sizes were measured using a caliper at 0, 1, 3, 5, and 7 days following wound. Changes in wound areas over time were expressed as the percentage of the initial wound areas. Also, wound area was digitally photographed using a digital camera in red box (3 × 3 cm) (Canon ES350, Ohta-ku, Tokyo, Japan). 

### 2.4. Immunohistofluorescence Staining

At 7 days post-wound, the rats were sacrificed and the skin was removed for histological examination. Skin tissue samples were fixed in 10% formalin for 24 h before embedding in paraffin. The blocks were cut into 5 *μ*m section in order to perform hematoxylin and eosin (H&E) and immunohistostaining. The sections were dewaxed in histoclear (Sigma, St. Louis, MO, USA) and rehydrated through a graded alcohol series. After retrieval (Abcam, Cambridge, MA, USA), the sections were blocked with normal goat serum for 1 h at room temperature. The sections were incubated at 4°C overnight with the following antibodies: mouse antiproliferation cell nuclei antigen (PCNA; Millipore, Billerica, MA, USA), rabbit polyclonal antibody to CD-31 (CD-31; Abcam), Collagen type I (Abcam), *α*-smooth muscle (*α*-SMA, Abcam). After washing, sections were incubated in Alexa 546-conjugated goat anti-mouse IgM (Molecular probe, Eugene, Oregon, USA) and Cy2-conjugated goat anti-rabbit IgG (Jackson ImmunoResearch) for 1 h at room temperature. After washing, the sections were counterstained with 4′,6-diamidino-2-phenylindole (DAPI; Sigma-Aldrich). Fluorescent images were acquired using a fluorescence microscope equipped with a spot digital camera (Nikon, Chiyoda-ku, Tokyo, Japan) and a Zeiss LSM 510 confocal scanning laser microscope (× 200 oil objective) (Carl Zeiss, Jena, Gemany). 

To determine PCNA and PCNA labeled-CD-31, *α*-SMA, and collagen type I-positive cells, every fifth coronal section per animal was prepared and counting was performed on three randomly selected non-overlapping per section. The measurement was made in a predefined field (300 *μ*m × 300 *μ*m) and the number of positive cells of wound area were obtain by multiplying by three. Using Meta-Morph imaging program (Molecular Devices Inc, Downingtown, PA, USA), wound area was determined by counting PCNA, CD-31, *α*-SMA, and collagen type I-positive cells.

### 2.5. Enzyme-Linked Immunosorbent Analysis

At 2 and 7 days following wound, wound samples were homogenized in t-per tissue protein extraction buffer (Pierce, Rockford, IL, USA) with protease inhibitor. The lysates were cleared by centrifugation (10,000 g) for 30 min at 4°C and the supernatant was kept at −70°C. The supernatant was examined using the enzyme-linked immunosorbent assay (ELISA) to detect the protein levels of angiogenic factor and inflammatory cytokines. The supernatant was further analyzed to quantify the concentration of VEGF (R&D system, Minneapolis, USA), TNF-*α* (R&D system), and IL-1*β* (R&D system) in strict accordance with the manufacturer's protocols.

### 2.6. Statistical Analysis

The behavior tests, cerebral ischemic volume, and cell count of apoptotic cells for both rat groups were subjected to one-way ANOVA with post hoc analysis, independent *T*-test, or Mann-Whitney *U* test. Data are presented as the mean value ± standard deviation of the mean. Probability values less than 0.05 were considered statistically significant.

## 3. Results

### 3.1. Wound Area

**Figure 2:**
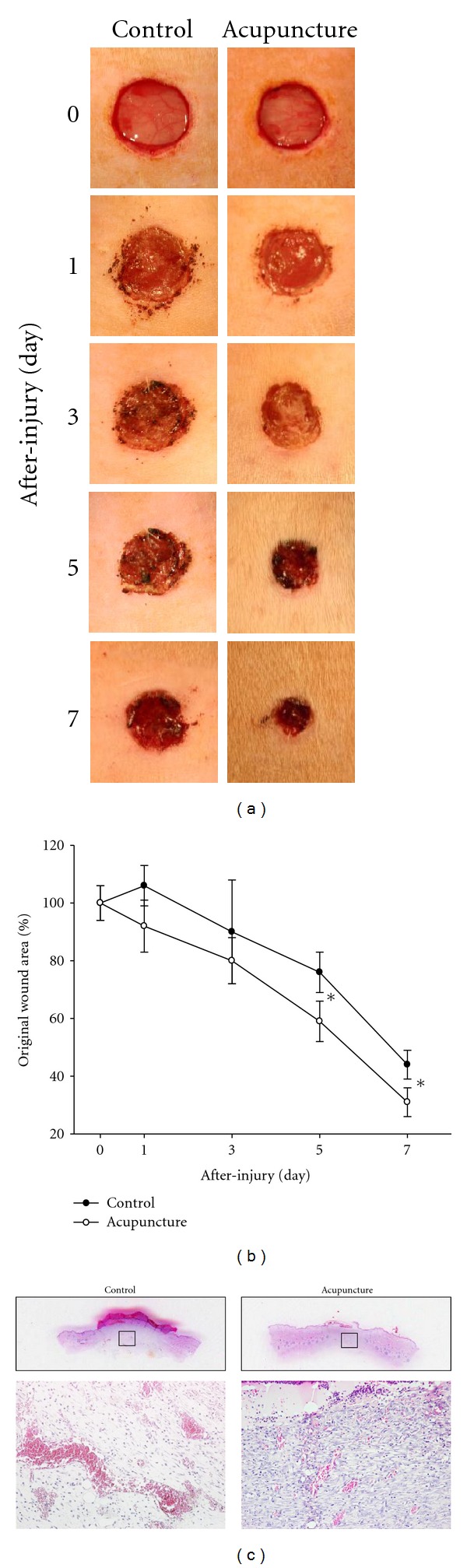
Wound closure in wound rat. Image shown in the wound image of acupuncture and control group at 0, 1, 3, 5, and 7 days after-wounding (a). Wound area was measured at the indicated time points of after-wounding (b). Area of the wounds was determined by quantitative analysis using caliper. Cutaneous wounds at 7 days were stained with (H&E) and photographed with a digital camera mounted on a light microscope (c). The wound sizes of acupuncture-treated group significantly decreased compared to the control group. Also red blood cells within the number of blood vessels decreased compared to the control group. Data are expressed as mean ± SD, *P < 0.05, Scale bars denote, 100 *μ*m.

Wound sizes were measured at 0, 1, 3, 5, and 7 days post-wounding. Wound closure was noted to progress more rapidly in acupuncture-treated group compared to the control group (Figure 2). At 7 days post-wounding, the wound sizes of acupuncture-treated group significantly decreased compared to the control group (31 ± 5 versus 44 ± 5%, *P* < 0.05). These results suggest that acupuncture can accelerate the restoration of wound healing. 

### 3.2. Expression of Inflammatory Cytokine

**Figure 3:**
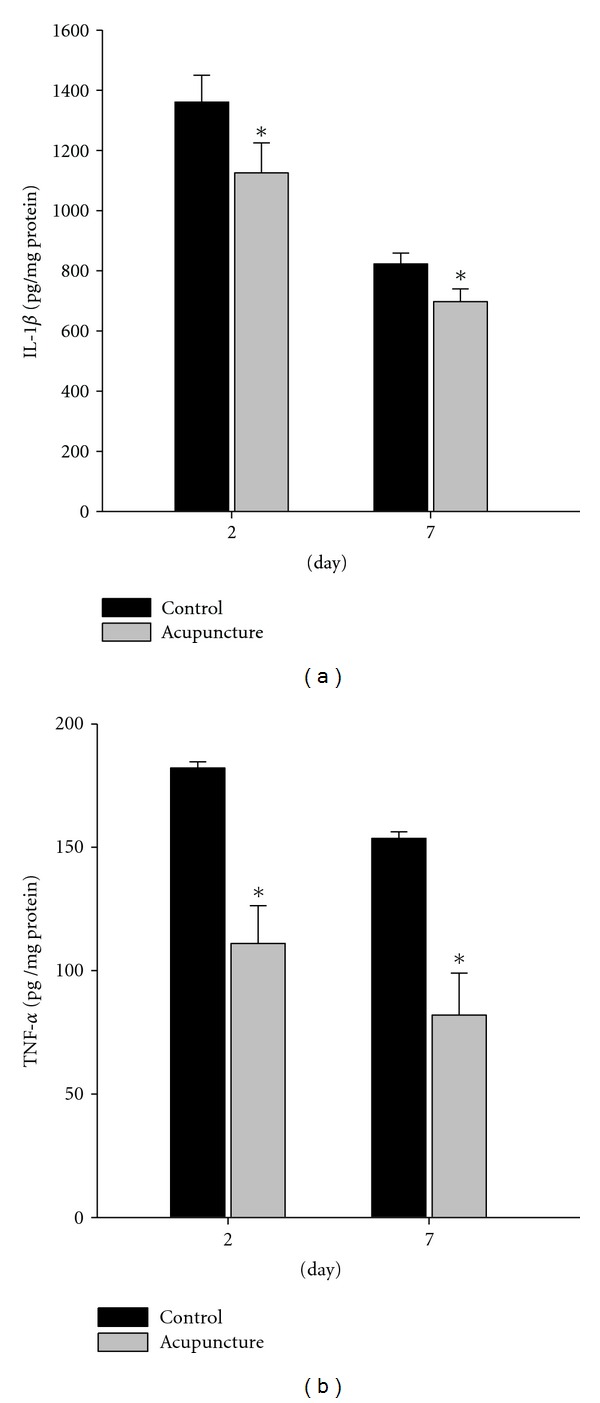
Expression of inflammatory cytokines in wound area. TNF-*α* and IL-1*β* were detected by ELISA at days after-wound. Protein levels of these factors show the quantitative analysis data (a-b). TNF-*α* and IL-1*β* were significantly decreased compared with the control group (a-b). Data are expressed as mean ± SD, *P < 0.05.

We hypothesized that acupuncture may protect an injured area following a wound by reducing the production of inflammatory cytokines. We used the ELISA to detect protein levels of inflammatory cytokines at 2 and 7 days in the wound area. At 2 and 7 days post-wounding, pro-inflammatory cytokines such as IL-1*β* and TNF-*α* were significantly reduced compared to the control group (Figure 3). These results suggest that acupuncture could promote wound healing to regulate inflammatory cytokines. 

### 3.3. Endogenous Cell Proliferation

**Figure 4:**
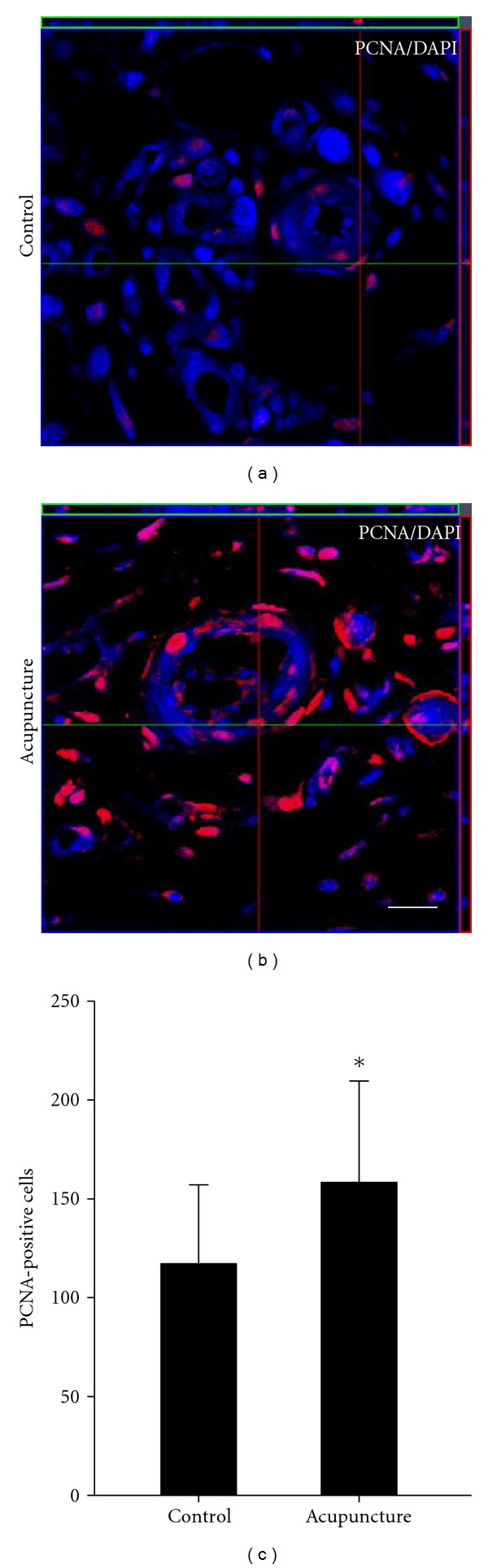
Quantitative analysis of PCNA-labeled cells in the wound area. Cell proliferation was measured by immunostaining using anti-PCNA antibody. At 7 days after-wounding, PCNA-labeled cells were present in wound area (a-b). PCNA positive cells were counted. In acupuncture-treated group, proliferation of endogenous cell was significantly increased compared to the control group (c). Data are expressed as mean ± SD, *P < 0.05, Scale bars denote, 50 *μ*m.

To investigate whether acupuncture treatment improved newly generated cell during wound healing, the number of proliferating cells were determined by anti-PCNA antibody. At 7 days after wound, proliferation of the newly generated cells increased greatly in acupuncture treated group compared to the control group within the wound area (Figure 4). These results suggest that acupuncture could have potentially improved cell proliferation during wound healing.

### 3.4. Angiogenesis

**Figure 5:**
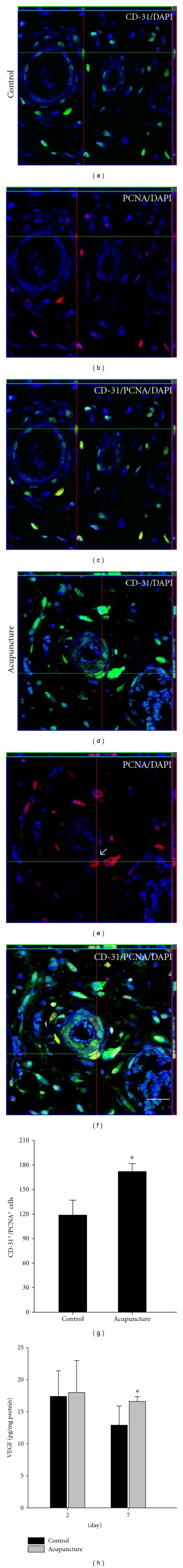
Quantitative analysis of angiogenesis by acupuncture in wound area. Histological analysis was shown into PCNA and CD-31 staining at 7 days after wound (a–f). The numbers of PCNA/CD-31-labeled cells were quantified present in wound area (g). At 7 days after-wounding, the expression of VEGF was significantly increased compared to the control group (h). Data are expressed as mean ± SD, *P < 0.05, Scale bars denote, 50 *μ*m.

To investigate whether acupuncture treatment promotes angiogenesis, angiogenesis factor, and endothelial cell of newly generated cells such as VEGF and CD-31 were analyzed using the ELISA and immunostaining. The VEGF is produced by a variety of cell types during wound healing, and is a potent stimulator of proliferation and migration in endothelial cells [15–17]. The expression of VEGF was significantly increased compared to the control group at 7 days after wound (16.6 ± 0.7 versus 12.9 ± 2.9 pg/mg, *P* < 0.05) (Figure 5(h)). This suggests that acupuncture may enhance angiogenesis of newly generated cells. In acupuncture-treated group, PCNA labeled CD-31-positive cells were increased compare to the control group in the wound area (172 ± 9.6 versus 118 ± 18.2, *P* < 0.05) (Figure 5(g)). These results suggest that the acupuncture could effectively promote angiogenesis by increasing VEGF expression in wound model.

### 3.5. Epidermal Regeneration

**Figure 6:**
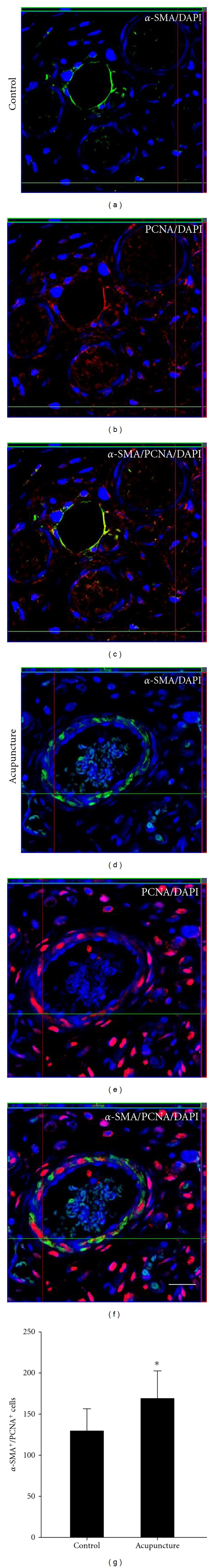
Quantitative analysis of epidermal regeneration by acupuncture in wound area. Histological analysis was shown into PCNA and *α*-SMA staining at 7 days after wounding (a–f). The numbers of PCNA/*α*-SMA-labeled cells present were quantified in wound area (g). At 7 days after-wounding, the PCNA/*α*-SMA-labeled cells were decreased compared to the control group. Data are expressed as mean ± SD, *P < 0.05, Scale bars denote, 50 *μ*m.

**Figure 7:**
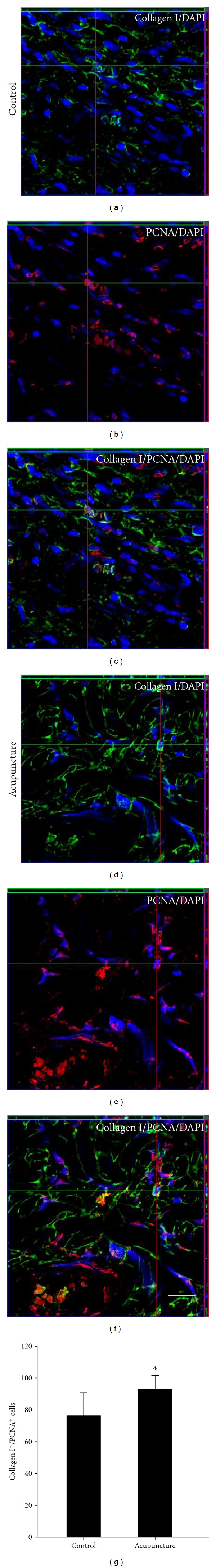
Quantitative analysis of epidermal regeneration by acupuncture in wound area. Histological analysis was shown in PCNA and collagen type I staining at 7 days after wounding (a–f). The numbers of PCNA/collagen type I-labeled cells present were quantified in wound area (g). At 7 days after-wounding, the PCNA/collagen type I-labeled cells were increased compared to the control group. Data are expressed as mean ± SD, *P < 0.05, Scale bars denote, 50 *μ*m.

We examine whether acupuncture could promote the induction of extracellular matrix remodeling by immunostaining for PCNA and several phenotype markers including *α*-SMA and collagen type I in the wound area. At the wound edge, vascular smooth muscle cells were analyzed using *α*-SMA antibody. The expression of *α*-SMA was observed at vascular smooth muscle cells of subcutaneous tissue (Figures 6(a) and 6(d)). In acupuncture-treated group at 7 days post-wounding, the PCNA labeled *α*-SMA-positive cells were significantly increased compared to the control (169 ± 33 versus 129 ± 26, *P* < 0.05) (Figure 6(g)). Also, PCNA labeled collagen type I-positive cells were significantly increased compared to the control (93 ± 8 versus 76 ± 14, *P* < 0.05) (Figure 7). These results suggest that the acupuncture may be associated with mechanisms involved in stimulating wound healing through increasing extracellular matrix protein such as *α*-SMA and collagen type I.

## 4. Discussion

The aim of wound healing is to promote rapid wound closure and recover functional properties. In this study, we examined the effect of the acupuncture treatment on wound model such as healing of the wound area, expression of inflammatory cytokines, cell proliferation, angiogenesis, and granulation tissue formation. 

Acupuncture has a long history as an Asian medical procedure that was used to treat various diseases such as inflammatory diseases, complex regional pain syndromes, and neurological diseases [18]. Previously studies reported therapeutic effect of acupuncture on Ashi-point in pain, brun, and bone fracture disease [12–14]. Also they suggested that acupuncture could have effects on wound healing process through inhibition of inflammatory cytokines, improvement of proliferative cells, and stimulation of epidermal regeneration. Therefore, we suggest that acupuncture treatment around the edges of wound might improve healing process. In this study, we observed wound healing at 1, 3, 5, and 7 days. The acupuncture-treated group showed a more reduced compared to the control group. 

Also, we analyzed in the pro-inflammatory cytokines such as TNF-*α* and IL-1*β* in the early phases of wound healing using the ELISA. We demonstrated that the expression of TNF-*α* decreased in acupuncture-treated group compared to the control at 2 and 7 days. Also, the expression of IL-1*β* was decreased compared to control group. These results suggest that acupuncture stimulation may have protective effects in wound model through the suppression of pro-inflammatory cytokines, which are secreted by macrophages [19, 20]. 

We investigated whether the acupuncture treatment could promote proliferation of newly generated cells and enhance the angiogenesis and granulation formation of newly generated cells by performing immunostaining for PCNA and several phenotype markers including CD-31, *α*-SMA, and collagen type I. PCNA-positive cells were counted at the edge of the wound at 7 days after wound model. In the acupuncture treated group, PCNA-positive cells were significantly increased compared to the control group. Also, we evaluated angiogenesis of newly generated cells by counting PCNA and CD-31-positive cells. PCNA-labeled CD-31 cells were increased in acupuncture group compared to the control at 7 days. Further, we observed the expression levels of VEGF at 2, and 7 days after wound model. The expression of VEGF was also increased compared to the control group at 7 days. The increase of expression of angiogenesis factors over time might be related to the acceleration of wound healing. 

Granulation-tissue formation and contraction are fundamental steps during the wound healing process, which can be analyzed by observing newly generated *α*-SMA and collagen type I. The expression of *α*-SMA was observed not only at vascular smooth muscle cells of subcutaneous tissue but also at myofibroblasts around granulated area in connective tissue. Also, increase in the deposition of collagen type I, the major collagen type in skin, is consistent with a more organized and stronger repaired skin [21]. Furthermore, collagen type I play an important role guiding keratinocytes and dermal fibroblasts migration in the wounded area. In acupuncture treated group, the PCNA labeled *α*-SMA and collagen type I-positive cells were significantly increased compared to the control. These results suggest that acupuncture may be associated with the wound healing effect by increasing extracellular matrix proteins. 

In conclusion, the result of our study suggests that acupuncture treatment around the edges of wound promotes wound healing through decreasing inflammatory cytokine release, increasing newly generated cells, and stimulating angiogenesis and granulation-tissue formation. Further studies are needed to identify the precise mechanism of action behind acupuncture.
